# Neutron imaging for magnetization inside an operating inductor

**DOI:** 10.1038/s41598-023-36376-x

**Published:** 2023-06-06

**Authors:** Hiroaki Mamiya, Yojiro Oba, Noriki Terada, Kosuke Hiroi, Tadakatsu Ohkubo, Takenao Shinohara

**Affiliations:** 1 National Institute for Materials Science, Tsukuba, 305-0047 Japan; 2 Japan Atomic Energy Agency, Tokai, 319-1195 Japan; 3 Present Address: Toyohashi University of Technology, Toyohashi, 441-8580 Japan

**Keywords:** Electrical and electronic engineering, Electronic devices, Characterization and analytical techniques, Imaging techniques

## Abstract

Magnetic components are key parts of energy conversion systems, such as electric generators, motors, power electric devices, and magnetic refrigerators. Toroidal inductors with magnetic ring cores can be found inside such electric devices that are used daily. For such inductors, magnetization vector*** M*** is believed to circulate with/without distribution inside magnetic cores as electric power was used in the late nineteenth century. Nevertheless, notably, the distribution of ***M*** has never been directly verified. Herein, we measured a map of polarized neutron transmission spectra for a ferrite ring core assembled on a familiar inductor device. The results showed that ***M*** circulates inside the ring core with a ferrimagnetic spin order when power is supplied to the coil. In other words, this method enables the multiscale operando imaging of magnetic states, allowing us to evaluate the novel architectures of high-performance energy conversion systems using magnetic components with complex magnetic states.

## Introduction

**Figure 1:**
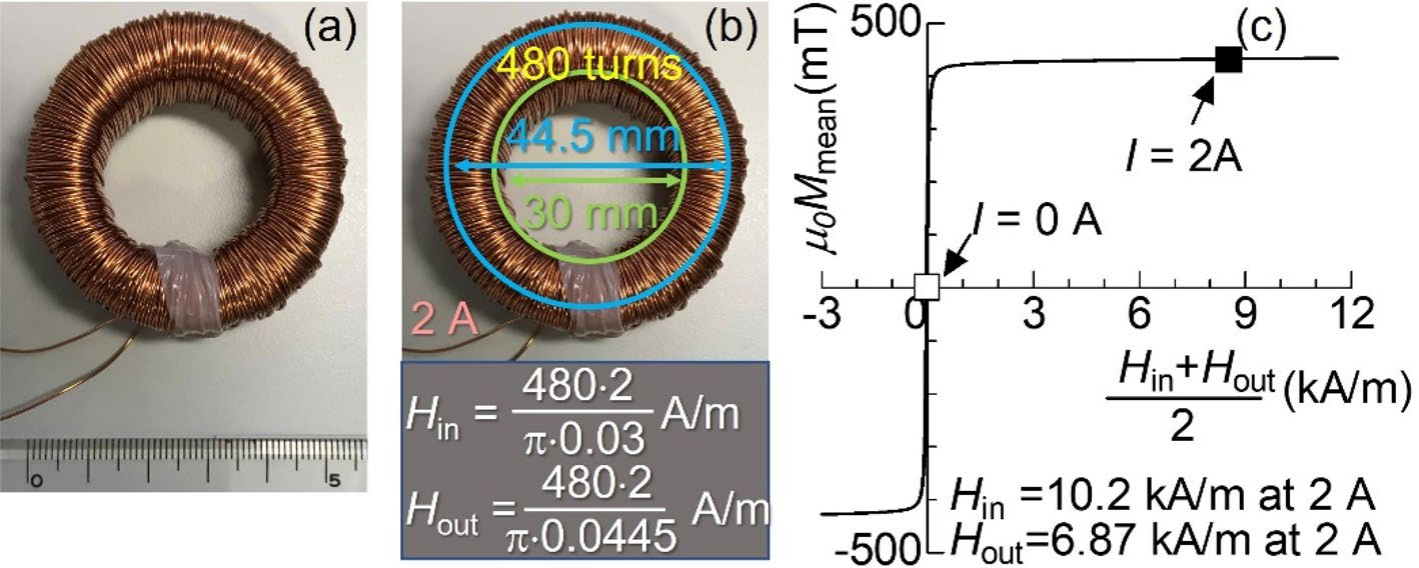
Toroidal inductor with manganese a zinc ferrite ring core. (**a**) Photograph prior to the measurement. According to Ampère’s circuital law, the amplitude *H* at the inner and outer peripheries, *H*_in_ and *H*_out_, is calculated in (**b**). (**c**) Average magnetization is shown as a function of average magnetic fields at 296 K. The square symbols in (**c**) denote the points for measuring neutron transmission spectra.

Magnetic components are key parts of energy conversion systems, such as electric generators, motors, power electric devices, and magnetic refrigerators. Therefore, they have been a mainstay of modern society since the late nineteenth century^1^. For example, toroidal inductors with ferrite cores and copper coils (Fig. 1a) are found in various electric devices in everyday life. According to Ampère's circuital law, a circumferential magnetic field ***H*** is generated inside an inductor coil when an electric current is supplied to the inductor^1^. Ampère’s circuital law predicts that the amplitude of *H* in the inner circumference is 1.5 times greater than that in the outer one because of the difference in perimeters (Fig. 1b). If magnetization ***M*** is induced in a direction parallel to ***H*** and its magnitude is proportional to *H, ****M*** also goes around circumferentially inside the ferrite core with 1.5 times different magnitudes between the inner and outer sides. Although such simple assumptions are not always valid for actual magnetic components used in energy conversion systems, the distribution of ***M*** inside it has never been directly verified. Because of magnetic saturation, nonlinear magnetic responses are frequently expected in a homogeneous large* H*. Moreover, demagnetizing fields generated at the corners or magnetic anisotropy tilt the direction of ***M*** form ***H*** in general magnetic components. In other words, actual magnetic components do not satisfy the well-known relationship with the following magnetic flux ***B*** = *μ*_0_(***H*** + ***M***) = *μ*_0_(1 + *χ*)***H***, where *μ*_0_ is the vacuum permeability and *χ* is the susceptibility. Thus, the distribution of ***M*** is not the same as the distribution of ***H*** or ***B***. However, over the centuries, magnetic components have been designed using only information from magnetization curves averaged as a whole (Fig. 1c) because the distribution of ***M*** cannot be measured unless disassembling the system.

Currently, magneto–optical Kerr microscopy or spin-polarized scanning electron microscopy is used to elucidate the fine distribution of ***M*** on the bare surface of bulky magnetic components^2^, whereas the ***H*** distribution outside the component can be accurately measured using fluxgate or Hall magnetic sensors. On the other hand, the means to non-destructively observe the distributions inside bulky components assembled in energy conversion systems have not yet been established. For example, ***H*** inside the magnetic core was approximated to the measured value using a search coil placed inside holes drilled into the core^3^. Consequently, we usually infer the internal distributions of ***H*** and ***M*** by comparing the surface or outside information with electromagnetic simulations^4,5^. This indirect evaluation worked for current designs using existing simple magnetic components. However, future sustainable societies will require a higher efficiency of energy conversion enabled by highly sophisticated magnetic components, where ***M*** or its original spin orientations are designed to be nonparallel and nonproportional to ***H*** at multiscale. For example, microscopically, some spins are antiparallel to ***H*** orientations (so-called ferrimagnetic) in a permanent magnet of (Nd_1−x_Dy_x_)_2_Fe_14_B^6^, soft magnet of (Mn_1−x_Zn_x_)Fe_2_O_4_^7^, magnetocaloric material of ErCo_2_^8^, and spintronic material of GdFeCo^9^, where the antiparallel spins play an important role in their magnetic performance. The tilted spins in the soft-magnetic phase contribute to increasing the energy product in exchange-spring composite magnets at the mesoscale^10^. Macroscopically, functionally graded magnetic materials^11^ and multimaterial components^12^ are used, where the magnetic properties are designed to vary from one place to another inside a single component unit. The magnetic components must be complex on multiscales. It is difficult to evaluate the internal distributions of ***H*** and ***M*** (otherwise ***B*** and ***M***) in such advanced materials using the information obtained from the surface or outside. The lack of a useful evaluation method is a barrier to the advancement of energy conversion systems.

Neutrons are highly penetrative compared with electrons and X-rays. Additionally, the spins of neutrons exhibit precession motion around ***B***, and some of the neutrons are scattered by electron spins that are microscopic ***M***. Therefore, imaging techniques for the precession of the initially polarized neutrons have been developed recently to visualize the distribution of ***B***^13–15^. Meanwhile, neutron scattering has played a critical role in the fundamental study of complex spin arrangements for a long time, where diffractometry detects scattered neutrons with high accuracy. In this context, the ***M*** distribution can be easily visualized using neutron diffractometry. However, neutrons scattered at different positions intersect each other when the scattering angles are not the same (See supplementary information). Alternatively, narrowed incident beams must be scanned for such mapping using diffractometry. Consequently, this method is time consuming. However, when such scattering occurs, the intensity of transmission neutrons decreases. This type of decrease observed in the neutron transmission spectra is known as Bragg edges^16,17^. Recently, we have demonstrated that spin arrangements can be evaluated by analyzing Bragg edges^18^, indicating that the simultaneous mapping of internal spin arrangements in a wide area is possible using a pair of parallel large-diameter rectilinearity beams and a two-dimensional (2D) detector because the straight trajectories of transmitted neutrons are not crossed over. Herein, we used neutron transmission spectroscopy to evaluate the potential value for imaging of ***M*** and microscopic spin arrangements inside magnetic components. We verified the distribution of ***M*** and microscopic spin arrangements inside the inductor (Fig. 1) as the simplest magnetic component suitable for the first experiment.

## Results and discussion

**Figure 2:**
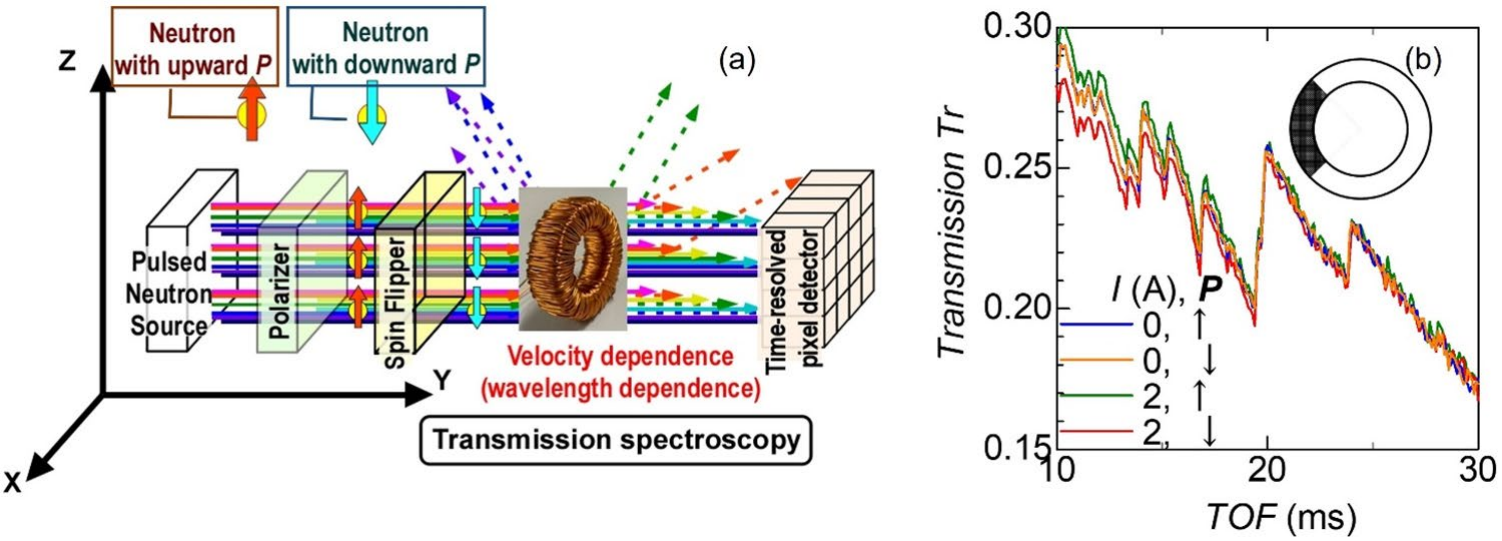
Transmissions *Tr* of the test inductor with manganese zinc ferrite ring core for neutrons with upward/downward polarizations as functions of the time of flight (TOF) of neutrons. (**a**) shows the schematic chart of the transmission experiment, and (**b**) show the results obtained in the standby state with a 0 A current *I* and the operating state with a 2 A current *I*, where *Tr* is averaged in the coloured part in the inset.

**Figure 3:**
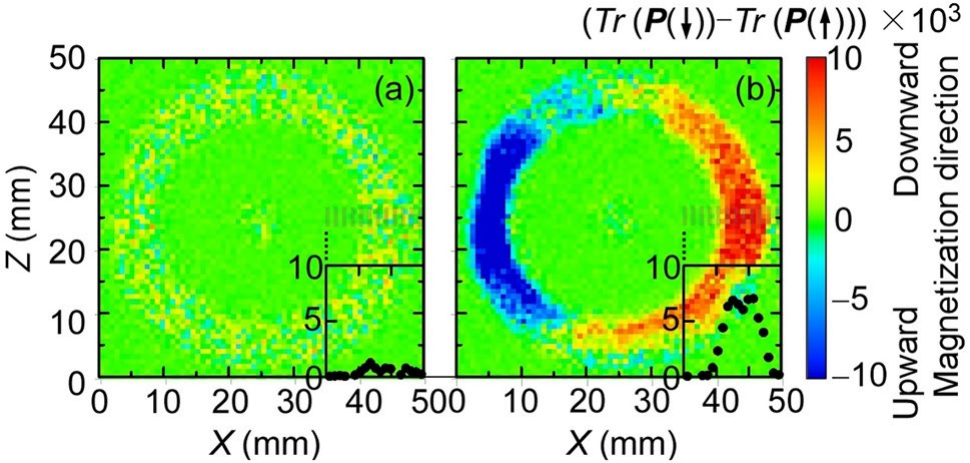
Contour maps of the difference in *Tr* for *P*(↓) to that for *P*(↑) in each pixel of the detector in (**a**) the standby state and (**b**) the operation state. The insets show the difference along a horizontal line at the vertical centre on the right. (the grey striped region).

Figure 2 shows the neutron transmissions *Tr* of the left part of the test inductor with manganese zinc (Mn–Zn) ferrite ring core as a function of the time of flight (TOF) of neutrons. In this figure, the standby state represents the 0 A current (*I*) to the coil and the operating state represents the 2 A current, corresponding to the remanent and almost saturated states respectively (Fig. 1c). The polarization vector of the incident neutron*** P*** was kept upward ***P***(↑) or rotated downward*** P***(↓) by turning the neutron spin flipper off or on^19^ (Fig. 2a). It is observed that as TOF increases, *Tr* gradually decreases with edge-like fine structures. *Tr* for neutrons with ***P***(↑) and*** P***(↓) differs considerably in the operating state, whereas they are the same in the standby state. In general, magnetic scattering at j-th atom is constructive for nuclear scattering at the same atom when ***P*** is antiparallel to ***m***_⊥j_ and destructive for nuclear scattering when ***P*** is parallel to ***m***_⊥j_, where ***m***_⊥j_ = (*m*_⊥_^x^_j_, *m*_⊥_^y^_j_, *m*_⊥_^z^_j_) is the vector projection of the magnetic moment ***m***_j_ = (*m*_xj_, *m*_yj_, *m*_zj_) of j-th atom on the plane perpendicular to the scattering vector, ***q***. As stated above, the variation in the intensity of transmission has an inverse relationship with that in the total magnitude of scatterings with variously oriented ***q*** at the atoms. Therefore, the result that *Tr* is smaller for ***P***(↓) than that for ***P***(↑) can be attributed to the state that the sum of ***m***_j_, i.e., ***M*** is primarily directed upward through the transmission path (details are analysed later). Conversely, when *Tr* for ***P***(↓) exceeds that for ***P***(↑), we can consider the downward direction of ***M***. The contour maps of the difference in *Tr* for ***P***(↓) to that for ***P***(↑) in each pixel of the detector, where *Tr* is averaged from 10 to 30 ms TOF is shown in Fig. 3. In the operating state, *Tr* in the left part of the test inductor decreases, as ***P*** changes from ***P***(↑) to*** P***(↓), whereas *Tr* in the right part of the test inductor increases with the change (Fig. 3b), indicating that when ***H*** is generated,*** M*** is directed upward in the left part and downward in the right part. However, ***M*** in the top and bottom parts of the test inductor seems not direct neither upward nor downward. These results are consistent with the simple model, where ***M*** circulates in a clockwise direction inside the ferrite ring core during the operating state. The insets in Fig. 3 show the difference in *Tr* for ***P***(↓) to that for ***P***(↑) along a horizontal line at the vertical centre on the right (the grey striped region). In the operating state, the difference is almost constant in the ranges where the ring core exists. In brief, the magnitude ***M*** is constant inside the ferrite core. For the ring core, the perimeter of the outer circle is 1.5 times longer than that of the inner one. Consequently, the amplitude *H* at the inner periphery is 1.5 times larger than that at the outer one because of the difference in the wire number density (Fig. 1b). If ***M*** is proportional to ***H***, the magnitude of ***M*** induced on the inner side of the core must be 1.5 times greater than that on the outer side. Alternatively, when the core is magnetically saturated, the magnitude of ***M*** is almost constant over the entire core. The feature observed here seems consistent with the latter case. This is reasonable because the magnetization curve is almost saturated when *H* is applied (Fig. 1c). The current analysis shows that ***M*** circulates peripherally inside the ring core with almost equivalent magnitudes between the inner and outer sides in contrast to the naïve prediction based on the relationship ***M*** = *μ*_0_*χ****H***. Let us examine edge-like fine structures of the obtained spectra to elucidate the microscopic magnetic states next.

The neutron transmission is represented by an exponential decay $Tr = e^{ - A\left( \lambda \right)}$, where *A*(*λ*) is the absorbance. For the multicomponent test inductor, *A*(*λ*) is expressed as the sum of every attenuation effect^17^:

1 $$A(\lambda ) = \sum\limits_{{\text{i}}} {\left( {\sigma_{{\text{Bragg,i}}}^{{{\text{ela}}}} + \sigma_{{\text{diffuse,i}}}^{{{\text{ela}}}} + \sigma_{{\text{incoh,i}}}^{{{\text{ela}}}} + \sigma_{{\text{i}}}^{{{\text{inela}}}} + \sigma_{{\text{i}}}^{{{\text{abs}}}} } \right)} n_{{\text{i}}} t_{{\text{i}}}$$

where *i* represents the ferrite core (Fe), copper wire (Cu), and resin coating (Re). The elastic Bragg scattering cross section $\sigma_{{{\text{Bragg}},{\text{i}}}}^{{{\text{ela}}}}$, elastic diffuse one $\sigma_{{{\text{diffuse}},{\text{ i}}}}^{{{\text{ela}}}}$, elastic incoherent one $\sigma_{{{\text{coh}},{\text{ i}}}}^{{{\text{ela}}}}$, inelastic one $\sigma_{{\text{i}}}^{{{\text{inela}}}}$, and absorption cross section $\sigma_{{\text{i}}}^{{{\text{abs}}}}$, contribute for each component, *n*_i_ and *t*_i_ represent the total number of unit cells in the unit volume and the effective thickness of the i-th component, respectively. Among these terms, only the Bragg scattering for the periodic structures of the nucleus at the position of (*x*_j_,* y*_j_,* z*_j_) and magnetic moments ***m***_j_ form fine structures in the spectra because the Laue condition is satisfied at a specific *λ*. In polycrystalline materials, such Bragg scattering occurs at various *λ* that are less than twice the interplanetary spacing, *d*_hkl_, of the {*hkl*} planes. Consequently, we can observe an edge-like structure for absorbance, known as the Bragg edge, at 2*d*_hkl_^17^. For thermal neutrons, the other terms have monotonous dependencies on *λ*; hence, they are discussed in the supplementary section, and we shall concentrate on $\sigma_{{{\text{Bragg}},{\text{i}}}}^{{{\text{ela}}}}$ described as follows:

2 $$\sigma_{{{\text{Bragg}},{\text{i}}}}^{{{\text{ela}}}} = \frac{{\lambda^{2} }}{{2v_{0} }}\sum \left[ {\left( {F_{{\text{N}}} \left( {hkl} \right) + F_{{\text{M}}}^{{{\text{nsp}}}} \left( {h^{\prime } k^{\prime } l^{\prime } } \right)} \right)^{2} + \left( {F_{{\text{M}}}^{{{\text{sp}}}} \left( {h^{\prime\prime }k^{\prime\prime }l^{\prime\prime }} \right)} \right)^{2} } \right]d_{hkl} R_{hkl} P_{hkl} E_{hkl} ,$$

where *v*_0_ is the unit cell volume^2,17^. The complementary error function, March–Dollase orientation distribution function, and Sabine’s primary extinction function were used for the resolution function* R*_hkl_, the preferred orientation function* P*_hkl_, and primary extinction function* E*_hkl_ with crystallite size *R*_c_, respectively. The crystal and magnetic structure factors for nonspin flip and spin-flip scatterings of neutrons, *F*_N_(*hkl*), *F*_M_^nsf^(*h*′*k*′*l*′), and *F*_M_^sf^(*h*″*k*″*l*″) are expressed as follows:

3a $$F_{{\text{N}}} \left( {hkl} \right) = \mathop \sum \limits_{j} o_{{\text{j}}} b_{{\text{j}}} {\text{exp}}\left[ {2{\pi i}(hx_{j} + ky_{j} + lz_{j} )} \right]{\text{exp}}\left[ {\frac{{ - B_{{{\text{iso}}}} }}{{4d_{hkl}^{2} }}} \right]$$

3b $$F_{{\text{M}}}^{{{\text{nsf}}}} \left( {h^{\prime } k^{\prime } l^{\prime } } \right) = \mathop \sum \limits_{j} o_{{\text{j}}} \left( { \mp r_{{\text{m}}} \frac{{m_{ \bot j}^{{{z}}} }}{{2\mu_{B} }}f_{j} } \right){\text{exp}}\left[ {2{\pi i}(h^{\prime } x_{j} + k^{\prime } y_{j} + lz^{\prime }_{j} )} \right]\exp \left[ {\frac{{ - B_{{{\text{iso}}}} }}{{4d_{h^{\prime}k^{\prime}l^{\prime}}^{2} }}} \right],$$

3c $$F_{{\text{M}}}^{{{\text{sf}}}} \left( {h^{\prime\prime }k^{\prime\prime }l^{\prime\prime }} \right) = \mathop \sum \limits_{j} o_{{\text{j}}} \left( { - r_{{\text{m}}} \frac{{m_{ \bot j}^{{{x}}} \pm im_{ \bot j}^{y} }}{{2\mu_{B} }}f_{j} } \right){\text{exp}}\left[ {2{\pi i}(h^{\prime\prime }x_{j} + k^{\prime\prime }y_{j} + l^{\prime\prime }z_{j} )} \right]\exp \left[ {\frac{{ - B_{{{\text{iso}}}} }}{{4d_{h^{\prime\prime }k^{\prime\prime }l^{\prime\prime }}^{2} }}} \right],$$

where *r*_m_ is the factor of magnitude (5.39 fm);* B*_iso_ is the temperature factor; and *o*_j_, *b*_j_, and *f*_j_ are the site occupancy, nucleus scattering length, and magnetic form factor of the j-th atom, respectively^2^. The negative and positive signs in Eq. (3b) correspond to the cases for neutrons with upward polarized states ***P***(↑) and with downward state*** P***(↓), respectively.

**Figure 4:**
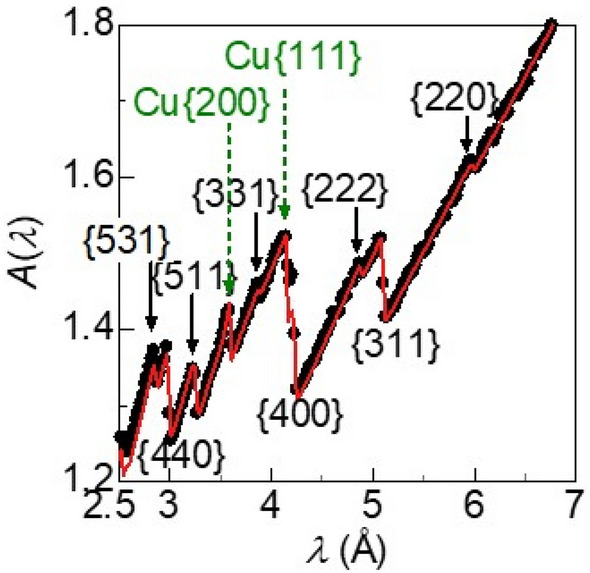
Wavelength dependence of the absorbance *A*(*λ*) in the standby state using the 0 A current: the black circles show the observed results, and the red curve shows the fitted line. {*hkl*} shows the diffraction planes of Mn–Zn ferrite and Cu.

Let us begin the investigation of the edge-like fine structures with analysing the transmission spectra in the standby state using the 0 A current *I* (Fig. 4). Because the spectrum is invariant when ***P*** is rotated from upward ***P***(↑) to downward*** P***(↓) by the neutron spin flipper, macroscopic ***M*** does not direct either upward or downward directions. Additionally, we cannot find extra Bragg edges compared with the cubic-spinel structure of ferrite and the FCC structure of copper. Therefore, the magnetic structure is collinear ferrimagnetic, as previously observed for Mn_1−x_Zn_x_Fe_2_O_4_ ferrites (*x* < 0.5): {*h*′*k*′*l*′} = {*h*″*k*″*l*″} = {*hkl*}^20^. For the ferrite core, we assume that all Zn ions occupy the tetrahedral interstitial site (A site) of the cubic-spinel structure^21^ and that each crystallite is randomly oriented: *P*_hkl_ = 1. Furthermore, *B*_iso_ for the ferrite and copper is set at 0.4 Å^2^^21^. The observed absorbance can be well reproduced with the curve calculated using Eqs. (1–3) (Fig. 4), and the parameters are shown in Table 1, where the agreement factor *R* is 0.3% for the edge width of 0.02 Å (see details in the supplementary section) The distribution ratio of Mn ions of the A site to octahedral (B) site is 0.267–0.124, which can be compared with that in a previous report^22^. The magnetic moment ***m***_B_ averaged over the ions of Mn_0.062_Fe_0.938_ at the B site is 1.6 μ_B_, and it is antiparallel to the magnetic moment ***m***_A_ of − 1.4 μ_B_ averaged over the ions of Zn_0.498_Mn_0.267_Fe_0.235_ at the A site. The total magnetic moment for a unit chemical formula is roughly 2 μ_B_, which is almost the same as the value estimated from the bulk magnetization at room temperature (see details in the supplementary section). We can confirm that the microscopic magnetic states in the standby state agree with the previously reported results for bare Mn–Zn ferrites that have not yet been assembled in an inductor device^20–23^. In the subsequent section, we will consider how the ferrite core becomes microscopically magnetized when an exciting current is supplied to the copper wires.

**Table 1:** Crystalline and magnetic parameters obtained by fitting for the observed *A*(*λ*).

Component	Ferrite core	Copper wire	Resin coating
Crystal structure	Cubic-spinel	FCC	–
Thickness *t*_i_ (mm)	13.0*	2.5	0.42
Lattice constant (Å)	8.46	3.59	–

A site composition	Zn_0.498_Mn_0.267_Fe_0.235_	A site magnetic moment *m*_A_	− 1.4 μ_B_
B site composition	(Mn_0.062_Fe_0.938_)_2_	B site magnetic moment *m*_B_	1.6 μ_B_
Oxygen position *u*	0.387	Crystallite size *R*_c_	11.5 μm

*The thickness *t*_Fe_ was fixed at a known value of 13.0 mm.

As discussed above, there are variations in the transmission at the right and left parts of the test inductor when ***P*** was rotated from upward ***P***(↑) to downward*** P***(↓) using the neutron spin flipper in the operating state with the exciting current (Fig. 2). Looking the spectra in more details, variations of some Bragg edge heights are considerable, while those of the other are insignificant. Considering Eqs. 2 and 3, such differences between the edges come from variety of $F_{{\text{M}}}^{{{\text{nsf}}}} \left( {h^{\prime } k^{\prime } l^{\prime } } \right)$ with positive and negative signs in the term of $\left( {F_{{\text{N}}} \left( {hkl} \right) + F_{{\text{M}}}^{{{\text{nsp}}}} \left( {h^{\prime } k^{\prime } l^{\prime } } \right)} \right)^{2}$. In other words, we can, in principle, estimate the magnitude and sign of $m_{ \bot j}^{{{z}}}$ of j-th atom in each crystalline site from the analyses on fine variations of the spectra between ***P***(↑) and*** P***(↓). For simplicity, we shall concentrate on the variations at Bragg edges because ***q*** at the edges *λ* = 2*d*_hkl_ becomes antiparallel to the incident direction (the backscattering). In the case, ***m***_⊥j_ is just written as (*m*_xj_, 0, *m*_zj_) for neutrons from the Y axis as ***q*** is given by (0, − *q*_y_, 0). Consequently, the difference in *A*(*λ*) between neutrons with ***P***(↑) and*** P***(↓) is expressed as follows:

4 $$\Delta A\left( { = 2d_{hkl} } \right){ }\sim \frac{{n_{{{\text{Fe}}}} t_{{{\text{Fe}}}}\lambda^{2} }}{{2v_{0} }}4F_{{\text{N}}} \left( {hkl} \right)F_{{\text{M}}}^{{{\text{nsf}}}} \left( {hkl,m_{ \bot j}^{{\text{z}}} = m_{\text{z}j} } \right)d_{hkl} P_{hkl} E_{hkl} ,$$

at *λ* = 2*d*_hkl_.

In addition to these scatterings, the precession motion around ***B*** occurs, as stated earlier. Therefore, we must also consider this effect. In general soft-magnets, ***M*** is expected to be parallel to ***B*** on average: 〈***M***〉//***H***. On the condition, ***B*** is expressed as μ_0_(***H*** + 〈***M***〉) + μ_0_*Δ****M***, where *Δ****M*** is the local fluctuation of ***M***. If *Δ****M*** is negligible, the polarization ***P*** starts to process around μ_0_(***H*** + 〈***M***〉) after the neutrons enter the ferrite core. During the precession motion, the angle between ***P*** and ***H*** + 〈***M***〉 is constant. Consequently, the angle between microscopic*** m***_j_ and ***P*** is also preserved because of the nature of ***M***//***m***_j_ in collinear ferrimagnets. In other words, the above discussion on scattering cross sections is applicable even if the precession motion occurs.

Finally, we shall consider the case that effects of locally fluctuated *Δ****M*** are not negligible. As known as the depolarization effect, numerous tiny random rotations around inhomogeneous μ_0_*Δ****M*** cause the reduction of the length of ***P***. In previous studies, the reduction rate per unit transmission path is described as $\alpha = cR_{f} (\mu_{0} \Delta M)^{2} \lambda^{2}$, where *c* = 2.15 × 10^29^ m^−4^ T^−2^ is a constant, relating the Larmor precession and *R*_f_ is the typical size of the local magnetic inhomogeneities, respectively^24^. In this analysis, the depolarization effect was considered because it was observed in a Mn–Zn ferrite even when the magnetization is almost saturated^25^. Because this type of depolarization is dominant, Eq. (4) has been revised.

5 $$\Delta A\left( { = 2d_{hkl} } \right){ }\sim \frac{{n_{{{\text{Fe}}}} t_{{{\text{Fe}}}} }\lambda^{2}}{{2v_{0} }}\left[ {2P_{0} \left( {\alpha t_\text{Fe} } \right)^{ - 1} \left( {1 - e^{{\alpha t_\text{Fe} }} } \right)} \right]F_{{\text{N}}} \left( {hkl} \right)F_{{\text{M}}}^{{{\text{nsf}}}} \left( {hkl,m_{ \bot j}^{{\text{z}}} = m_{\text{z}j} } \right)d_{hkl} P_{hkl} E_{hkl} ,$$

where the details are discussed in the supplementary section. In the evaluation of the magnitude and sign of ${m}_{\text{z}j}$, *R*_c_ estimated in the above analyses is used as* R*_f_ because the prior study on polycrystalline Mn–Zn ferrites showed that *R*_f_ is almost identical to the grain sizes^26^. In addition, we set *ΔM*^2^ at *M*_s_^2^ − *M*_z_^2^, where the saturation magnetization* M*_s_ is estimated using the law of approximation to magnetic saturation and *M*_mean_ at *I* = 2 A is used as *M*_z_ (see the supplementary section). Because *F*_N_(*hkl*), *P*_hkl_, and *E*_hkl_ are unalterable by the current, we can use them as estimated in the standby state. We estimate *m*_zj_ at each pixel by fully analysing the variations in the Bragg edge heights in the {311}, {511}, {440}, and {531} planes of the spinel ferrite, where the spin arrangement is assumed collinear as observed in the standby state because it was reported that the collinear structure holds in Mn_1−x_Zn_x_Fe_2_O_4_ ferrites (*x* < 0.5) at the high magnetic field of 4 MA/m^20^.

**Figure 5:**
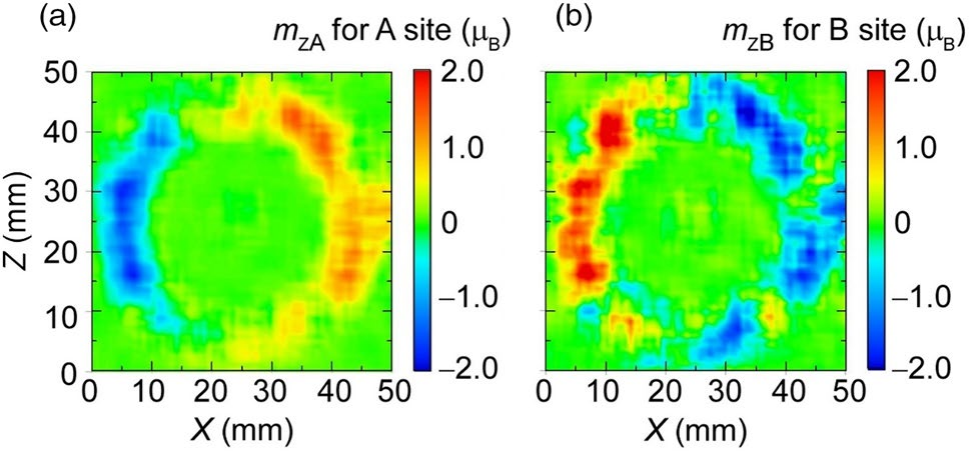
Distribution of microscopic magnetic state. (**a**) shows the Z-component of magnetic moment, ${\varvec{m}}_{{\text{A}}}$ estimated for ions in A site and (**b**) exhibits Z-component of ${\varvec{m}}_{{\text{B}}}$ in B site, where they are averaged in the region of 5 × 5 pixels.

The estimated *m*_zj_ of the ions at A and B sites,* m*_zA_ and *m*_zB_, are mapped in Fig. 5. The magnitude of *m*_zA_ is ~ 1 μ_B_ and its sign is positive in the right part of the ferrite ring core, indicating that the direction of ${\varvec{m}}_{{\text{A}}}$ of A-site ions in the right part is in the upper hemisphere, whereas ${\varvec{m}}_{{\text{A}}}$ of A-site ions in the left part of the ring core is directed in the lower hemisphere because of the estimated negative sign of $m_{{\text{zA}}}$ in the part. These results are consistent with the interpretation that the direction of ${\varvec{m}}_{{\text{A}}}$ rotates anticlockwise inside the ferrite ring core during the operating state, against the clockwise circulation of macroscopic ***M*** (Fig. 3). Conversely, the sign of *m*_zB_ is negative in the right parts and positive in the left part. These show that ${\varvec{m}}_{{\text{B}}}$ of B-site ions directs downward and upward in the right and left parts, respectively. In other words, the direction of ${\varvec{m}}_{{\text{B}}}$ appear to rotate clockwise, as seen for macroscopic ***M***. The observed antiparallel relationship between ${\varvec{m}}_{{\text{A}}}$ and ${\varvec{m}}_{{\text{B}}}$ is reasonable because it has been known that ${\varvec{m}}_{{\text{B}}}$ is antiferromagnetically coupled with ${\varvec{m}}_{{\text{A}}}$ on the microscopic scale^22^. Notably, the result obtained here seemed qualitatively invariant with respect to the detailed conditions of the assumption, although many assumptions were employed as stated above.

## Summary and prospective

We described the magnetic states inside an inductor device using a Mn–Zn ferrite ring core because no study has directly validated the internal distribution of magnetization vectors ***M*** even in such simple inductors. In the experiment, polarized neutron transmission spectra were measured at each pixel of a 2D time-resolved detector. The results showed that ***M*** circulates in the clockwise direction inside the ring core when power is supplied to the coil. Detailed analyses on the edge-like fine structures in the spectra can also clarify the microscopic magnetic state and its distribution. For example, magnetic moments in the A-site orient in the direction opposite to that of macroscopic ***M****.* The results obtained here are consistent with the simple prediction for the inductor device using a Mn–Zn ferrite ring core, as expected for over a century. This is the first direct verification of the prediction for the magnetic core and an indication that polarized neutron transmission spectroscopic imaging has potential to image the distribution of the macroscopic magnetic state in highly sophisticated magnetic devices with complex structures. We also hope that material/site selective magnetic imaging derived from the edge-like fine structures would be helpful in evaluating the distributions of microscopic magnetic states not only in a homogeneous ferrimagnet employed for the demonstration here but also in functionally graded materials or in materials unevenly cooled/ pressed during a bulky manufacturing process.

However, there remain many issues in the current stage. In respect of the spatial resolution, it is important to selectively use the neutron transmission spectroscopic imaging and other imaging techniques such as X-ray magnetic imaging according to the size of measuring object. In the generally used energy range, the penetration depth of X-ray on magnetic materials is less than 1 mm. Therefore, we expect the neutron magnetic imaging to measure the object thicker than 1 mm. If a minimum spatial resolution of 1% is essential, it corresponds to 0.01 mm for the parts measuring a few millimetres unit per side. The actual spatial resolution obtained herein, 1 mm or more (see Fig. 3), is apparently insufficient for the purpose. Thus, further progress on detectors with high spatial resolutions is necessary for the experiments on smaller objects. Meanwhile, the maximum size depends on the penetration depth of neutron on the measuring object. Considering the actual transmission was 20–30% for the present inductor with a total thickness of 15 mm (Fig. 2), the upper limit of total thickness is a few tens of millimetres for the magnetic devices (not containing neutron absorbing elements such as B, Cd, and Gd). Therefore, this technique is suitable for evaluating small- to medium-sized power conversion devices. With respect to time resolution, it is presently possible to measure only steady states and their periodic responses that can be stroboscopically imaged, as evidenced by the fact that 36 ks was needed for the present measurement. The observations of time-dependent variations such as aging effects require improving the counting rate of the detector because we could not effectively use the powerful pulsed neutron source in J-PARC to avoid miscounting in the detector (see “Materials and Methods” section). With respect to evaluating microscopic states, further advances in analysis methods for the depolarization effects would be highly required to estimate the magnitude of the magnetic moments with high accuracy. Finally, we must mention that it is inevitable that irradiated objects are more or less radio-activated. Especially, it is difficult to bring back the magnetic devices containing elements with high neutron activations such as Co and Eu even after a year. As discussed here, neutron transmission spectroscopic imaging has many issues to be resolved and it is still inconvenient to apply to the evaluation of a wide variety of novel magnetic components with complex magnetic states at the multiscale; however, we can say that this new method is worth developing further because the evaluation of such complex magnetic states designed by novel architectures will be essential for improving the performance of next generation energy conversion systems.

## Materials and methods

Manganese zinc (Mn–Zn) ferrite core was commercially supplied by TDK Co. and used as the main component of the test inductor, where the outer and inner diameters of the ring core, thickness, and density are 44.5, 30.0, 13.0 mm, 5000 kg/m^3^, respectively. Inductively coupled plasma emission analysis shows that the compositional ratios of Mn, Zn, and Fe are 0.13:0.166:0.704, respectively. The *M–H* loop was measured using a B–H analyzer (SY8219, IWATSU Electric Co). The coil was made by winding polyester resin-coated copper wire with a 0.5-mm diameter on the ferrite core with 480 turns (Fig. 1).

The neutron transmission spectra of the inductor were measured as functions of the TOF at beamline 22 (BL22) RADEN in J-PARC^27^. A magnetic mirror was used to polarize a 50 × 50 mm neutron beam. Consequently, the polarization *P* = (*I*_+_ − *I*_−_)/(*I*_+_ + *I*_−_) became approximately *P*_off_ ~ 0.9, where *I*_+_ and *I*_−_ are the intensities of neutrons in spin-up and spin-down states^19^. Subsequently, the polarities of neutron spins were reversed by a gradient- RF type neutron spin flipper. Because the efficiency of the spin flipper was very close to one, the polarization changed to *P*_on_ ~  − 0.9. These polarized states were maintained using guide magnets until the neutrons entered the inductor. A time-resolved neutron gas electron multiplier n-GEM 2D-detector with an effective detection area of 100 × 100 mm and pixels of size 0.8 × 0.8 mm was used to count the transmitted neutrons at a distance of 18.5 m from the source. The effective peak count rate of the detector was 180 kcps; hence, the incident neutron flux density was reduced to the magnitude of 2 × 10^2^ n/s/mm^2^ in average to avoid miscounting in the detector. Consequently, the measuring time of each image was 36 ks. The velocity estimated from TOF was used to calculate *λ*. The experiments were performed in the standby state where no current *I* (0 A) was supplied to the coil and in the operating state with a current *I* of 2 A, where a magnetic field ranging from 6.87 to 10.2 kA/m was generated inside the coil (Fig. 1b).

## Supplementary Information

Supplementary Information.

## Data Availability

All data needed to evaluate the conclusions in the paper are present in the paper and/or the Supplementary Materials.
