# Supplementary material for: Neutron imaging for magnetization inside an operating inductor
Source: Sci Rep. 2023 Jun 6;13:9184. doi: 10.1038/s41598-023-36376-x (PMC10244398; doi:10.1038/s41598-023-36376-x)
Supplement: Supplementary file 1 — Supplementary Information. [file 41598_2023_36376_MOESM1_ESM.pdf]

Supplementary Text

Test inductor

For the magnetic core, we selected a manganese zinc ferrite with high permeability (H5C2), which is commercially supplied by TDK Co for transformer. In the material specification information, the initial permeability is 10000 ± 30%, the magnetic flux density at 1.2 kA/m is 0.4 T, the coercive force is 7.24 A/m, the Curie temperature is above 120°C, and the electrical resistivity is 0.15 Ω·m. The temperature dependence of magnetization for the ferrite was measured for an elongated rod-like shape sample using a SQUID magnetometer (Quantum Design MPMS3). Figure S1 shows the thermal variation of the total magnetic moment for a unit chemical formula, and it was found that with decreasing temperature below room temperature, it gradually increases and becomes 6.4 μ_B at 2 K. However, it decreases with the increase in temperature above room temperature and almost disappears at 400 K. This behaviour is consistent with the minimum Curie temperature of 120°C.

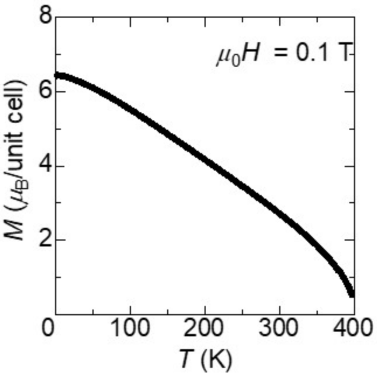

Fig. S1.
Thermal variation of the total magnetic moment of the manganese zinc ferrite (H5C2) for a unit chemical formula.

The M–H loop at room temperature was measured for a ring core of the manganese zinc ferrite (H5C2) using a B–H analyzer (SY8219, IWATSU Electric Co.). Fig. S2 shows the mean magnetization estimated from the induced voltage in the secondary coil as a function of the magnetic field averaged between the amplitudes at the inner and outer peripheries, H_{in} and H_{out}, where the measuring frequency is 300 Hz. We found that the magnetization is almost saturated when H is >1 kA/m. The saturation magnetization M_s was determined using the law of approach to saturation magnetization while considering appropriate high-field susceptibility χ_0, as shown in Fig. S2(b).

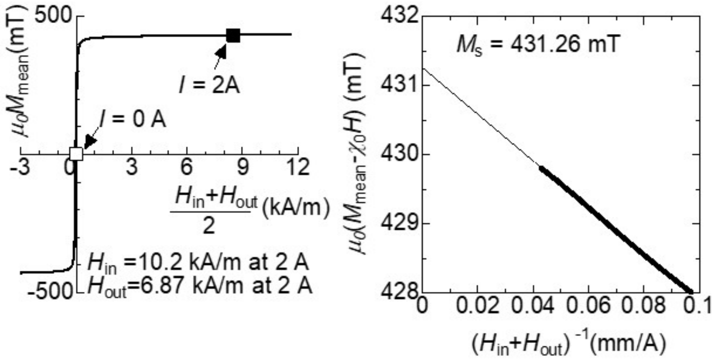

Fig. S2.

M–H loop for the manganese zinc ferrite H5C2 at room temperature. The right panel shows the mean magnetization as a function of the reciprocal of the average magnetic field to evaluate the saturation approach.

The shape of the core is a ring, and its outer and inner diameters and thickness are 44.5, 30.0, and 13.0 mm, respectively. The test inductor was made by winding a 0.5-mm copper wire on the ferrite core (480 turns). Consequently, the effective thickness of the copper wire layer was 0.55 mm. The inductor was cooled using a chiller device; however, the temperature of the inductor increased to 312 K (highest), as shown in Fig. S3, when an electric current *I* of 2 A was supplied.

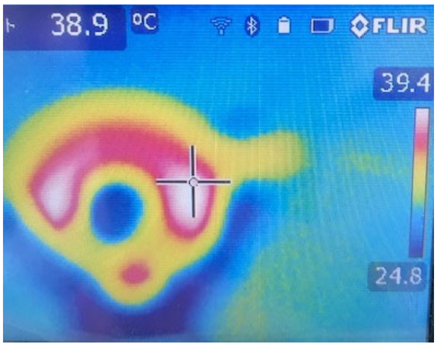

Fig. S3.
Thermal image of the test toroidal inductor with the ferrite ring core after a current of 2 A was supplied for 2 h.

Measurement principle

For neutron diffractometry, the paths of the neutrons were scattered at different positions across each other when the scattering angles were not the same, as shown in Fig. S4(a). In other words, the narrowed incident beams must be scanned one by one for such mapping by diffractometry. However, this method is highly time consuming. Nevertheless, when such scattering occurs, the transmission intensity of the neutrons decreases at the corresponding wavelength, as shown in Fig. S4(b). This type of decrease, as observed in the neutron transmission spectra, has been referred to as Bragg edges. This result indicates that the simultaneous mapping of internal spin arrangements in a wide area is possible using a pair of large-diameter parallel collimated beams from a pulsed neutron source and a two-dimensional time-resolved detector so that the straight trajectories of transmitted neutrons do not cross each other.

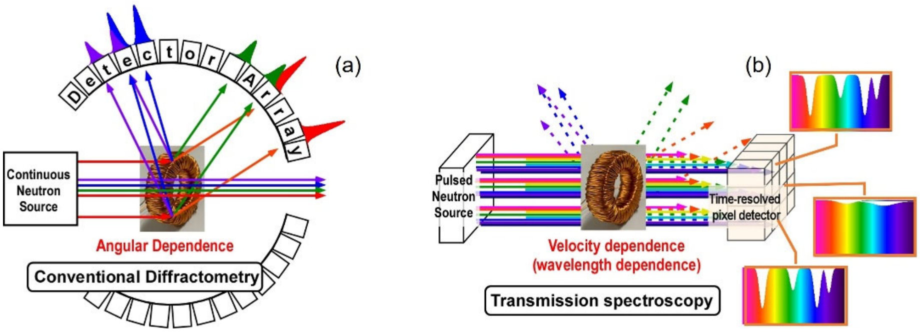

Fig. S4.
Schematic sketches of neutron imaging using conventional diffractometry and spectroscopy.

Absorbance and Bragg scattering in the Mn–Zn ferrite core

Although we mainly focused on the neutron cross section $\sigma^{\text{ela}}_{\text{Bragg,Fe}}$ caused by the Bragg scattering from the Mn–Zn ferrite core, notably, the neutron absorbance $A(\lambda)$ comes from various contributions [Ref. S1]:

$$A(\lambda) = \sum_i (\sigma^{\text{ela}}_{\text{Bragg,i}} + \sigma^{\text{ela}}_{\text{diffuse,i}} + \sigma^{\text{ela}}_{\text{incoh,i}} + \sigma^{\text{inela}}_{\text{i}} + \sigma^{\text{abs}}_{\text{i}}) n_i t_i, \tag{S1}$$

where i stands for the ferrite core (Fe), copper wire (Cu), and resin coating (Re). For each component, there are various contributions: elastic Bragg scattering cross section $\sigma^{\text{ela}}_{\text{Bragg,i}}$, elastic diffusion one $\sigma^{\text{ela}}_{\text{diffuse,i}}$, elastic incoherence one $\sigma^{\text{ela}}_{\text{coh,i}}$, inelastic one $\sigma^{\text{inela}}_{\text{i}}$, and absorption cross section $\sigma^{\text{abs}}_{\text{i}}$, where n_i and t_i represent the total number of unit cells in a unit volume and the effective thickness of the i-th component, respectively. The analysis provides $\sigma^{\text{ela}}_{\text{Bragg,Fe}}$, as shown in Fig. S5 and as described in the main text. Hence, we showed here the analyses of the other contributions.

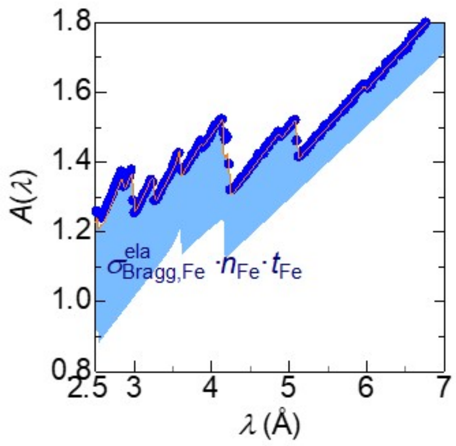

Fig. S5.
Total neutron absorbance of the inductor. The coloured region exhibits the contributions of the elastic Bragg scattering cross section $\sigma^{\text{ela}}_{\text{Bragg,Fe}}$ of the ferrite core (The details are discussed in the main text.)

Bragg scatterings from the copper wires

Fig. S6 shows $A(\lambda)$, which remained after deducting $\sigma^{\mathrm{ela}}_{\mathrm{Bragg,Fe}}$, which was estimated in the main text, as shown in Fig. S5. We could find that two edge-like structures remained at λ, corresponding to $2d_{\mathrm{hkl}}$ for the {111} and {200} planes of the FCC copper. Thus, we employed the conventional equation for the Bragg scattering, which is described as follows:

$$\sigma^{\mathrm{ela}}_{\mathrm{coh,Cu}} = \frac{\lambda^2}{2v_0}\sum\left[\left(F_{\mathrm{N}}(hkl)\right)^2\right] d_{hkl} R_{hkl} P_{hkl} E_{hkl}, \quad \text{(S2)}$$

where v_0 is the unit cell volume, and R_{hkl}, P_{hkl}, and E_{hkl} denote the resolution function, preferred orientation function and primary extinction function with a crystallite size of R_{c} [S1]. First, we simply assumed that all R_{hkl}, P_{hkl}, and E_{hkl} are unity and the shapes of the Bragg edges in Fig. S6 could not be well reproduced. Thus, we employed the March–Dollase orientation distribution function for P_{hkl}:

$$P_{\mathrm{hkl}} = \frac{1}{2\pi}\int_0^{2\pi}\left[G^2(\cos A\sin\theta_{\mathrm{hkl}} + \sin A\cos\theta_{\mathrm{hkl}}\sin\beta)^2 + \frac{1-(\cos A\sin\theta_{\mathrm{hkl}} + \sin A\cos\theta_{\mathrm{hkl}}\sin\beta)^2}{G}\right]^{-\frac{3}{2}} d\beta,$$

$$A = \arccos\left(\frac{hH+kK+lL}{\sqrt{h^2+k^2+l^2}\sqrt{H^2+K^2+L^2}}\right), \quad \text{(S3)}$$

where G is the preferred orientation parameter and <*HKL*> is the preferred orientation vector. The principal texture with the <*HKL*> of <110> and G of 0.57 accounts for 94% of the contribution, while the rest seems have a texture with a <100> and G of 0.34. The estimated t_{cu} was 2.5 mm, and this value is roughly consistent with a thickness of 1.1 mm, which was averaged for the copper wire with 480 turns.

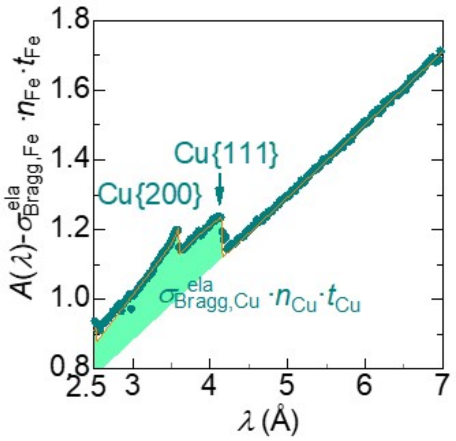

Fig. S6.

The absorbance $A(\lambda)$ that remained after deducting $\sigma_{\mathrm{Bragg,Fe}}^{\mathrm{ela}} \cdot n_{\mathrm{Fe}} \cdot t_{\mathrm{Fe}}$. The coloured region exhibits the contributions of the elastic Bragg scattering cross section $\sigma_{\mathrm{Bragg,Cu}}^{\mathrm{ela}}$ of the copper wires.

Elastic incoherent scatterings and absorption for the ferrite core and copper wires

The cross section resulting from the incoherent elastic scatterings was approximated as in [Ref. S1] as follows:

$$\sigma_{\mathrm{incoh,i}}^{\mathrm{ela}} \sim \sum_{j} 4\pi \left(\overline{|b_j|^2} - \overline{|b_j|}^2\right) \frac{\lambda^2}{2B_{\mathrm{iso}}}\left[1 - \exp\left(-\frac{2B_{\mathrm{iso}}}{\lambda^2}\right)\right],$$

where the temperature factor B_{iso} for the ferrite and copper was assumed to be 0.4 Å^2. The contribution of the variations of the nuclear spins, isotopes, and compositions $4\pi n_i \left(\overline{|b_j|^2} - \overline{|b_j|}^2\right)$ were set at 0.115 cm^{-1} and 0.046 cm^{-1} for $Mn_{0.39}Zn_{0.498}Fe_{2.11}O_4$ and the pure copper with natural isotopic abundances, respectively [Ref. S2]. The absorption cross sections, $\sigma_{\mathrm{abs,Fe}} n_{\mathrm{Fe}}$ and $\sigma_{\mathrm{abs,Cu}} n_{\mathrm{Cu}}$, were calculated to be 0.079 λ cm^{-1} and 0.178 λ cm^{-1}, respectively [Ref. S2].

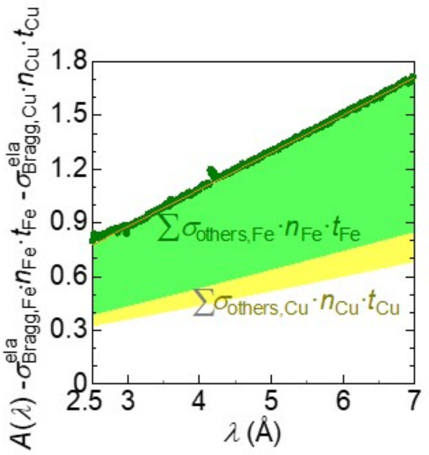

Fig. S7.

The absorbance $A(\lambda)$ that remained after deducting $\sigma_{\mathrm{Bragg,Fe}}^{\mathrm{ela}} \cdot n_{\mathrm{Fe}} \cdot t_{\mathrm{Fe}}$ and $\sigma_{\mathrm{Bragg,Cu}}^{\mathrm{ela}} \cdot n_{\mathrm{Cu}} \cdot t_{\mathrm{Cu}}$. The green and yellow coloured regions exhibit the contributions of the elastic incoherent scatterings and the absorption of the ferrite core and copper wires, respectively.

Total scattering cross section for the resin

Neutron attenuation in organic matter is mainly dominated by incoherent scatterings of hydrogen atoms. In a recent study [Ref. S3], the total cross section of an organic matter could be approximated as the sum of the terms of the average contributions of different functional groups, such as aliphatic (−CH), aromatic (−CH), methylene (=CH_2), and methyl groups (≡CH_3), thus neglecting their correlation. For an unsaturated polyester resin, the total cross section $\sigma_{\mathrm{incoh,H}}$ per one hydrogen atom was calculated, as shown in Fig. S8. We found that its λ-dependence is unfortunately different from the residual part $A(\lambda)-\sum_{i=\mathrm{Fe,Cu}}(\sigma^{\mathrm{ela}}_{\mathrm{Bragg,i}}+\ \sigma^{\mathrm{ela}}_{\mathrm{incoh,i}}+\sigma_{\mathrm{abs,i}})n_i t_i$. In other words, the residual part cannot be only explained by cross sections of resin, regardless of the resin thickness (t_{Resin}). The deviation between the residual part and $\sigma_{\mathrm{incoh,H}}$ seems significantly expanded at longer λ. Let us discuss the contribution that was not considered yet.

Elastic diffuse scattering cross section from magnetic fluctuations

At this stage, it should be noted that the electron spins in the ferrite are not completely ordered, as indicated by the difference in the magnetization between 2 K and the operating temperatures, as shown in Fig. S1. The difference δm is approximately 4 μ_B. Therefore, spin fluctuations with short-range correlations might cause additional contributions without Bragg edges. For the fluctuations parallel to the ordered magnetic moments, their correlation can be expressed as $\xi_0^{-2}/(\xi^{-2}+q^2)$ using the Ornstein–Zernicke formalism, where ξ_0 is the atomic interval size, and ξ is the correlation length. Assuming that $\xi \gg q^{-1}$ for the utilized λ and the minimum scattering angle θ_0 corresponding to the aperture angle of the detector, the cross section for magnetic diffuse scattering $\sigma^{\mathrm{ela}}_{\mathrm{diffuse,Fe}}$ was approximated to be $C\lambda^2$, where the proportional coefficient C is $\frac{1}{6\pi}\left(r_m \frac{\delta m}{2\mu_{\mathrm{B}}} f_j\right)^2 \xi_0^{-2}\ln(\tan(\theta_0/2))$. In this analysis, we took this contribution into account for explaining the total cross section. Consequently, the residual part $A(\lambda)-\sum_{i=\mathrm{Fe,Cu}}(\sigma^{\mathrm{ela}}_{\mathrm{Bragg,i}}+\ \sigma^{\mathrm{ela}}_{\mathrm{incoh,i}}+\sigma_{\mathrm{abs,i}})n_i t_i$ was reproduced when we considered both the incoherent scatterings of the resin and the elastic diffuse scattering of the magnetic fluctuations, where the former was calculated using the value of $\sigma_{\mathrm{incoh,H}}$ and a thickness t_{Resin} of 0.042 mm, and the latter was calculated using a proportional coefficient $\left(\frac{\delta m}{2\mu_{\mathrm{B}}}\right)^2 \xi_0^{-2}\ln(\tan(\theta_0/2))$ of 1.0 Å^{-2}. This result is acceptable, as the magnitudes of ξ_0 and $\ln(\tan(\theta_0/2))$ are 10^0 Å and 10^0, respectively. Here, it can be noted that, other than spin fluctuations, there are many candidates that can cause a contribution proportional to λ^2. However, they could not be distinguished in the observed transmission spectrum. Nevertheless, the critical point is that the results of the analysis of the Bragg edges for the fine structures of the spectrum, as described in the main text, were invariant, regardless of the selections of the origin of the λ^2 -dependence of the presently discussed part.

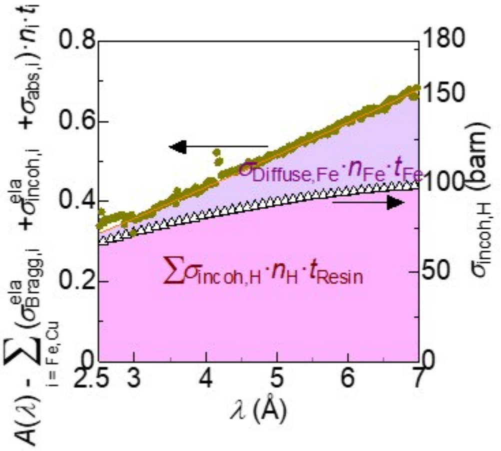

Fig. S8.

The absorbance $A(\lambda)$ that remained after deducting the above-mentioned contributions of the ferrite core and copper wires. The colored regions exhibit the contributions of incoherent scattering from the hydrogen in the resin and from the diffuse scattering of the spin fluctuations, respectively. The open triangles show the total cross section $\sigma_{\mathrm{incoh,H}}$ per one hydrogen atom calculated for an unsaturated polyester resin.

Approximated treatment of the depolarization and attenuation of the transmitted neutrons

When neutrons with spin-up (+) and spin-down (−) states are made incident from the Y-axis, each transmission intensity in the ferrite core is expressed as follows:

$$\frac{dI_{\pm}}{dy} = -\left\{ \sigma_{\mathrm{other}} + \frac{\lambda^2}{2v_0} \sum \left[\left(F_{\mathrm{N}} \mp \left| F_{\mathrm{M}}^{\mathrm{nsp}} \right| \right)^2 + \left(F_{\mathrm{M}}^{\mathrm{sp}} \right)^2 \right] d_{hkl} R_{hkl} P_{hkl} E_{hkl} \right\} n_{\mathrm{Fe}} I_{\pm} \pm D(\boldsymbol{B}, I_{+}, I_{-})$$

where $D(\boldsymbol{B}, I_{+}, I_{-})$ is the variation caused by the presession motion around $\boldsymbol{B}$ at the neutron location. We set $I = I_{+} + I_{-}$ and the amplitude of the polarization vector $\boldsymbol{P}$ as $P(y) = \frac{I_{+} - I_{-}}{I_{+} + I_{-}}$. Thus, the sum of both could be written as follows:

$$\frac{dI}{dy} = -\left\{ \sigma_{\mathrm{other}} + \sigma_{\mathrm{nonpol}} - 2P(y) \frac{\lambda^2}{2v_0} \sum \left[F_{\mathrm{N}} \left| F_{\mathrm{M}}^{\mathrm{nsp}} \right| \right] d_{hkl} R_{hkl} P_{hkl} E_{hkl} \right\} n_{\mathrm{Fe}} I(y)$$

$$\sigma_{\mathrm{nonpol}} = \frac{\lambda^2}{2v_0} \sum \left[(F_{\mathrm{N}})^2 + \left(F_{\mathrm{M}}^{\mathrm{nsp}} \right)^2 + \left(F_{\mathrm{M}}^{\mathrm{sp}} \right)^2 \right] d_{hkl} R_{hkl} P_{hkl} E_{hkl},$$

Since any presession motion does not change the total intensity; hence, the last terms cancelled each other out. We assumed that $P(y)$ can be described by an exponential decay $P_0 e^{-\alpha y}$ with a constant coefficient α. In this condition, we could execute the following integration:

$$I(y) = I_0 \exp\left(-\left\{ \left(\sigma_{\mathrm{other}} + \sigma_{\mathrm{nonpol}} \right) - \frac{\lambda^2}{2v_0} [2P_0 (\alpha y)^{-1} (1 - e^{\alpha y})] \sum \left[F_{\mathrm{N}}(hkl) \left| F_{\mathrm{M}}^{\mathrm{nsp}}(h'k'l') \right| \right] d_{hkl} R_{hkl} P_{hkl} E_{hkl} \right\} n_{\mathrm{Fe}} y \right)$$

References:

S1. H. Sato, "Quantitative Imaging of Crystalline Structure Information by using a Pulsed Neutron Transmission Method," thesis, Hokkaido University, Japan (2011).

S2. National Institute of Standards and Technology: https://www.ncnr.nist.gov/resources/activation/.

S3. G. Romanelli, D. Onorati, P. Ulpiani, S. Cancelli, E. Perelli-Cippo, J. I. M. Damián, S. C. Capelli, G. Croci, A. Muraro, M. Tardocchi, G. Gorini. Thermal neutron cross sections of amino acids from average contributions of functional groups J. Phys. Condens. Matter 33, 285901, (2021).
